# Correspondence of Basal Forebrain Resting‐State Functional Connectivity and Cerebral Glucose Metabolism Alterations With Neurotransmitter Maps in Alzheimer's Disease

**DOI:** 10.1002/cns.70901

**Published:** 2026-05-09

**Authors:** Yujie He, Hanxiao Xue, Sheng Bi, Yan Wang, Xiaocao Liu, Yixia Li, Zhigeng Chen, Dongdong Rong, Bixiao Cui, Jie Ma, Shaozhen Yan, Jie Lu

**Affiliations:** ^1^ Department of Radiology and Nuclear Medicine, Xuanwu Hospital Capital Medical University Beijing China; ^2^ Beijing Key Laboratory of Magnetic Resonance Imaging and Brain Informatics Beijing China; ^3^ Key Laboratory of Neurodegenerative Diseases Ministry of Education Beijing China

**Keywords:** ^18^F‐FDG PET, Alzheimer's disease, basal forebrain, functional connectivity, neurotransmitters

## Abstract

**Aims:**

To investigate alterations in basal forebrain (BF) subregional functional connectivity (FC), cerebral glucose metabolism, and their spatial correspondence with atlas‐based neurotransmitter distributions in Alzheimer's disease (AD).

**Methods:**

Forty‐two Aβ‐PET‐positive AD patients and forty‐one matched healthy controls (HC) underwent simultaneous PET/MRI. We analyzed resting‐state FC of BF subregions (Ch1‐3, Ch4) and measured cerebral glucose metabolism using ^18^F‐FDG PET standardized uptake value ratio (SUVR). Spatial correlations with neurotransmitter maps were assessed using the JuSpace toolbox.

**Results:**

Compared with HC, AD patients showed decreased FC between the left Ch4 and hippocampus/posterior cingulate gyrus, and increased FC between the right Ch4 and precentral/postcentral gyrus. Additionally, AD patients showed increased FC between the left Ch1‐3 and superior temporal gyrus/insula, decreased FC between the right Ch1‐3 and the orbitofrontal gyrus, and increased FC between the right Ch1‐3 and the left temporal lobe (voxel‐level *p* < 0.001, cluster‐level *p* < 0.05, GRF correction). These FC changes were spatially correlated with serotonergic (5HT1a, 5HT4, SERT) and dopaminergic (D1, D2, DAT) receptor distributions (*p* < 0.05, FDR corrected). Widespread cerebral hypometabolism in temporoparietal and frontal regions was spatially correlated with serotonin, dopamine, GABA, glutamate, and kappa‐opioid systems (*p* < 0.05, FDR corrected).

**Conclusion:**

The FC of BF and cerebral metabolic changes in AD show distinct spatial correspondence with specific neurotransmitter systems, highlighting the crucial involvement of serotonin and dopamine in AD pathophysiology.

## Graphical abstract

Altered functional connectivity of basal forebrain subregions (Ch1‐3, Ch4) and widespread cerebral hypometabolism were identified in Alzheimer's disease using simultaneous PET/MRI. Spatial correspondence with normative neurotransmitter receptor and transporter maps suggested that serotonergic and dopaminergic systems may contribute to AD‐related network vulnerability.

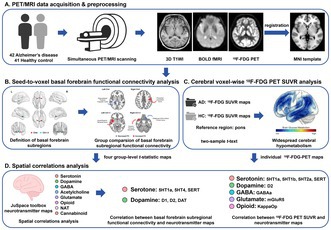

## 1 Introduction

Alzheimer's disease (AD) is the most common neurodegenerative disorder, characterized clinically by progressive memory impairment and cognitive decline, and pathologically by the accumulation of extracellular amyloid‐β (Aβ) plaques and intracellular neurofibrillary tangles composed of hyperphosphorylated tau protein [1, 2, 3, 4]. Dramatic loss of cholinergic neurons in the basal forebrain (BF) has a tight interrelation with early AD pathology and precedes widespread cortical degeneration [5, 6, 7]. As the principal source of cholinergic projection neurons, BF provides acetylcholine to widespread regions including the prefrontal cortex, hippocampus, entorhinal cortex, and amygdala, thereby modulating memory, attention, and cognitive functions [8, 9].

Growing neuroimaging evidence has supported the presence of BF degeneration and connectivity disturbances in AD [7, 10, 11, 12]. BF can be anatomically divided into four subregions including the medial septal nucleus (Ch1), the vertical limb of the diagonal band (Ch2), the horizontal limb of the diagonal band (Ch3), and the magnocellular complex (Ch4) [1], which mainly includes the Nucleus basalis of Meynert (NbM). Resting‐state fMRI studies have revealed altered intrinsic connectivity of the BF subregions and their correlations with cognitive performance, both in AD dementia [13, 14] and individuals with preclinical AD [15, 16, 17]. Additionally, Fluorine‐18‐fluorodeoxyglucose positron emission tomography (^18^F‐FDG PET) quantifies cerebral glucose metabolism using standardized uptake value ratio (SUVR) and is widely regarded as a reliable biomarker for evaluating neuronal metabolic activity in cognitive disorders [18]. Integrating resting‐state fMRI with ^18^F‐FDG PET facilitates simultaneous assessment of functional connectivity and neuronal metabolism, providing a more comprehensive understanding of circuit dysfunction and cognitive decline in AD.

In addition to resting‐state fMRI and ^18^F‐FDG PET, the role of neurotransmitters in neurodegenerative diseases is increasingly recognized as a critical dimension of understanding disease progression. Neurotransmitter systems, including the cholinergic, serotonergic, dopaminergic, glutamatergic, and GABAergic systems, are essential for neuronal communication [19]. Alterations in neurotransmitter synthesis, storage, transport, and degradation may result in neuronal dysfunction and contribute to AD‐related dementia [20]. Furthermore, emerging evidence from both clinical and animal studies has demonstrated the dysfunction of neurotransmitters in AD, particularly within the serotonergic [21, 22, 23], dopaminergic [24, 25, 26, 27], and GABAergic systems [28, 29, 30]. However, the integrated relationships among BF‐related FC changes, cerebral metabolic alterations, and neurotransmitter systems remain incompletely understood.

The JuSpace toolbox has recently been developed to examine spatial correlations between atlas‐based distributions of radioligands and other neuroimaging modalities [31]. This toolbox helps overcome technical constraints such as the high cost of PET and the limited availability of in vivo radioligands, which have restricted large‐scale case–control studies across receptor systems. Additionally, it has been utilized to investigate the spatial correspondence between neuroimaging alterations and neurotransmitter systems in various neurodegenerative disorders, including mild cognitive impairment (MCI) [32, 33], Parkinson's disease [34], and multiple sclerosis [35]. The multimodal integration of neurotransmitter receptor mapping with functional and metabolic parameters may offer new opportunities for understanding the neurochemical mechanisms underlying network disruption in AD and for developing targeted therapeutic strategies. In line with this framework, our previous study also identified neurotransmitter system dysregulation in AD, showing that the spatial distribution of cerebral glucose hypometabolism and Aβ burden was closely aligned with multiple neurotransmitter systems [36].

In this study, we aimed to characterize alterations in resting‐state FC of BF subregions (namely Ch1‐3 and Ch4) and cerebral glucose metabolism in patients with AD using simultaneous PET/MRI. We further employed the JuSpace toolbox to evaluate the spatial correlations between altered FC and ^18^F‐FDG SUVR and the atlas‐based distribution of specific neurotransmitters in AD. We hypothesized that BF subregional FC and cerebral glucose metabolic changes were spatially correlated with neurotransmitter receptor/transporter distributions, reflecting the neurochemical basis of AD‐related dysfunction.

## 2 Methods

### 2.1 Participants

We retrospectively recruited 42 clinically diagnosed ^18^F‐AV45 PET‐positive AD patients through the Neurodegenerative Disease Cohort between March 2022 and April 2024 at Xuanwu Hospital, Capital Medical University. The diagnosis of AD patients was established according to the National Institute on Aging‐Alzheimer's Association workgroups guidelines for probable AD and the revised criteria proposed by Jack et al. for the diagnosis and staging of AD in the presence of Aβ positive as confirmed by Aβ PET or cerebrospinal fluid examination [37, 38]. In this study, clinical assessment was performed by experienced neurologists, and biological confirmation of AD was achieved through the presence of neocortical Aβ deposition on simultaneous PET/MRI, as interpreted by specialized physicians in radiology and nuclear medicine.

The exclusion criteria for AD were as follows: (1) age < 45 years; (2) history of cerebrovascular diseases; (3) major psychiatric diagnoses including but not limited to severe depression, schizophrenia, and bipolar disorder; (4) neurological conditions unrelated to AD that could contribute to cognitive impairment, such as traumatic brain injury, neoplasms, or severe white matter lesions (Fazekas scores > 2 as assessed on T2‐FLAIR images); (5) poor imaging quality (head motion > 3 mm).

Forty‐one age‐ and sex‐matched healthy controls (HC) who had no history of neurological or psychiatric disorders, no head injuries or surgical operations, no abnormal signs on neurological examination, and no cognitive complaints with a Clinical Dementia Rating score of 0 were enrolled from the community for comparison.

### 2.2 Data Acquisition

Imaging data were obtained using the simultaneous time‐of‐flight (TOF) PET/MRI 3.0‐Tesla system (GE Healthcare, Waukesha, WI, USA) with a 19‐channel head–neck union coil for motion correction. The PET/MRI acquisition protocol was consistent with our previous studies [36, 39]. Each participant underwent ^18^F‐FDG PET, resting‐state fMRI, and high‐resolution three‐dimensional T1‐weighted imaging. In addition, AD patients received Aβ‐PET scan with ^18^F‐AV45 for diagnostic confirmation. The ^18^F‐FDG PET and Aβ‐PET scans were conducted within 7 days to prevent potential tracer interference and maintain optimal image quality.

Both ^18^F‐AV45 and ^18^F‐FDG radiotracers were synthesized by the Department of Nuclear Medicine, Xuanwu Hospital, Capital Medical University, with a radiochemical purity exceeding 95%. All participants underwent a 10‐min ^18^F‐FDG PET scan 50‐min after receiving an intravenous injection of 5.6–8.2 mCi of ^18^F‐FDG. Additionally, AD patients received an intravenous injection of 10 mCi of ^18^F‐AV45, followed by a 20‐min PET scan commencing 50‐min post‐injection. All subjects were instructed to remain still and keep eyes closed but not to fall asleep. Foam cushions were used to minimize head motion, and headphones were employed to mitigate scanner noise.

High‐resolution T1‐weighted images were collected using a sagittal 3D brain in volume (3D BRAVO) sequence with the following parameters: repetition time/echo time = 8.5/3.2 ms, flip angle = 15°, voxel size = 1 × 1 × 1 mm^3^, number of slices = 188. Resting‐state fMRI images were acquired using an echo‐planar imaging (EPI) sequence with a repetition time/echo time = 2000/30 ms, flip angle = 90°, voxel size = 3.59 × 3.59 × 4.40 mm^3^, number of slices = 33, 300 volumes, and interleaved slice acquisition. Both T1 and EPI sequences were corrected for B_0_ field inhomogeneity. PET emission data were acquired in 3D list mode, and a 2‐point Dixon scan (18 s) was performed for MR‐based attenuation correction. PET images were reconstructed using an iterative ordered‐subset expectation–maximization algorithm with time‐of‐flight and point‐spread function modeling (OSEM‐TOF‐PSF) with 8 iterations, 32 subsets and a 3 mm full‐width at half‐maximum Gaussian filter. The reconstructed PET image matrix was 192 × 192, field of view = 350 × 350 mm^2^, voxel size = 1.82 × 1.82 × 2.78 mm^3^ and spatial resolution = 4.5 mm.

### 2.3 fMRI Data Preprocessing

Resting‐state fMRI data preprocessing was performed using Data Processing Assistant for Resting‐State fMRI toolbox (DPARSFA, version 5.2; http://www.rfmri.org/DPARSF) [40], based on the Statistical Parametric Mapping version 12 (SPM12, Wellcome Centre for Human Neuroimaging, London, UK) in MATLAB R2024a platform (MathWorks, Natick, MA, USA), following procedures in our earlier studies [39]. The first 10 volumes were discarded because of transient signal fluctuations and scanner adaptation effects. The remaining images underwent slice‐timing correction for differences in acquisition timing across slices and were realigned using a six‐parameter rigid‐body transformation to correct for head motion. Participants with head motion exceeding 3.0 mm in translation or 3.0° in rotation were excluded. Individual T1‐weighted image was then co‐registered to the mean functional image and segmented into gray matter, white matter, and cerebrospinal fluid probabilistic maps using the SPM12 unified segmentation algorithm. Subsequently, functional data were spatially normalized to the MNI template, resampled into 3 × 3 × 3 mm^3^ and smoothed using a Gaussian kernel with a full width at half maximum (FWHM) of 6 mm. To control for potential confounding effects, nuisance covariates including the Friston 24‐parameter motion model, white matter, and cerebrospinal fluid signals were regressed out. Finally, temporal band‐pass filtering (0.01–0.1 Hz) was applied to minimize low‐frequency drifts and high‐frequency noise.

### 2.4 Seed‐Based FC Analysis

Based on its functional organization into two subregions [41], we defined seed regions corresponding to the anatomical divisions Ch1‐3 (medial septal/diagonal band nuclei) and Ch4 (nucleus basalis of Meynert) in both hemispheres (Figure S1). These seeds derived from probabilistic cytoarchitectonic maps implemented in the SPM12 Anatomy toolbox [42]. Specifically, each seed region was examined through co‐registration of fMRI and T1‐weighted images to minimize signal loss and mitigate potential artifacts. Then, seed‐to‐voxel FC analysis was performed by calculating the Pearson correlation between the mean time series of predefined seed regions and all voxels in the cerebrum, followed by Fisher's *z*‐transformation to generate *z*‐transformed FC maps for further statistical analysis.

### 2.5 PET Data Processing

All ^18^F‐FDG PET images underwent partial volume correction (PVC) using SPM12 to mitigate effects of cerebral atrophy, as reported in our previous studies [36, 39]. The PVC‐corrected images were then co‐registered to each participant's T1 images and subsequently normalized to the Montreal Neurological Institute (MNI) standard space. Non‐brain tissues and skull were removed to reduce potential noise. SUVR maps were generated using pons as the reference region for ^18^F‐FDG PET data. All images were smoothed using an isotropic Gaussian kernel with a FWHM of 8 mm.

### 2.6 Spatial Correlations Between FC, ^**18**^F‐FDG PET SUVR and Neurotransmitter Maps

The spatial correlations between BF FC, cerebral glucose metabolism alterations and neurotransmitter maps were estimated using the JuSpace toolbox (version 1.5; https://github.com/juryxy/JuSpace). This framework integrates voxel‐wise neuroimaging biomarkers with neurotransmitter maps from healthy cohorts, as previously described [31]. We analyzed a comprehensive set of neurotransmitter systems, including serotonergic (5HT1a, 5HT1b, 5HT2a, 5HT4, SERT), dopaminergic (D1, D2, DAT, FDOPA), glutamatergic (mGluR5, NMDA), GABAergic (GABAa), cholinergic (VAChT), noradrenergic (NAT), cannabinoid (CB1), and opioid (Kappa, MU) receptors/transporters see (Table S1).

### 2.7 Statistical Analysis

All statistical analyses were performed using SPSS 27.0 (IBM Corp., Armonk, NY, USA). Variable normality was first assessed using the Shapiro–Wilk test. Group comparisons between AD and HC were assessed using independent sample *t*‐tests for normally distributed continuous variables (or Mann–Whitney *U* tests if non‐normal) and chi‐squared tests for categorical variables. A two‐tailed *p* < 0.05 was considered statistically significant.

The seed‐based FC analysis of BF subregions was performed across the cerebrum. Individual *z*‐value maps were subjected to one‐sample *t*‐tests to identify brain regions exhibiting significant connectivity with each seed region (*p* < 0.001, false discovery rate (FDR) correction). Subsequently, two‐sample *t*‐tests were employed to compare between‐group FC differences using the statistical module in DPABI version 6.0, with age, sex, education years and head motion covariates included as nuisance regressors. The resulting group‐level *t* maps were thresholded at *p* < 0.001 corrected at voxel level and *p* < 0.05 corrected at cluster level for multiple comparisons using a Gaussian random field (GRF) method.

For voxel‐wise comparisons of ^18^F‐FDG PET SUVR maps between AD and HC groups, two‐sample *t*‐tests were conducted on the individual maps using SPM12 with age, sex and education years included as covariates. Statistical significance was determined at a voxel‐level threshold of uncorrected *p* < 0.001, and multiple comparisons were controlled using the Benjamini‐Hochberg FDR at *p* < 0.05.

Finally, we investigated whether the spatial patterns of BF subregional FC and cerebral glucose metabolism alterations in AD are associated with specific neurotransmitter systems using the JuSpace toolbox. For BF FC, we entered four group‐level *t*‐maps derived from the voxel‐wise comparisons between AD and HC for each BF subregion (left and right Ch1‐3, left and right Ch4; as described in Section 2.4) into JuSpace toolbox using option 4, and computed Spearman rank correlations between each *t* map and each receptor/transporter density map. For cerebral glucose metabolism, we used option 5 to calculate the Spearman correlation coefficients between voxel‐wise ^18^F‐FDG PET data from AD patients and HC and the neurotransmitter distribution maps included in the toolbox. The resulting correlation coefficients were converted to Fisher's *z* values for statistical evaluation. To test significance, the JuSpace toolbox implemented exact permutation testing (10,000 iterations with orthogonal label shuffling) to generate null distributions and evaluate whether the observed Fisher *z*‐transformed correlation coefficients differed significantly from zero. The Benjamini‐Hochberg FDR correction was used to control for multiple comparisons at *p* < 0.05.

## 3 Results

### 3.1 Demographic Characteristics

The demographic characteristics of the AD and HC groups are shown in Table 1. There were no significant group differences in sex and age (*p* > 0.05). Significant group differences were found in education years (*p* < 0.001), Mini‐Mental State Examination (MMSE) (*p* < 0.001), and Montreal Cognitive Assessment (MoCA) scores (*p* < 0.001). Compared with HC, AD patients showed significantly lower education years, MMSE scores and MoCA scores (*p* < 0.001) (Table 1).

**TABLE 1:** Demographic characteristics in AD and HC groups.

Characteristics	AD (*n* = 42)	HC (*n* = 41)	*t*/*χ* ^*2*^	*p*‐value
Sex (F/M)	27/15	27/14	0.022	0.881
Age (years)	63.29 ± 8.07	65.95 ± 5.99	−1.712	0.091
Education (years)	10.60 ± 4.15	13.56 ± 2.84	−3.791	< 0.001
MMSE	15.02 ± 6.24	29.34 ± 0.79	−14.737	< 0.001
MoCA	10.33 ± 5.10	26.63 ± 1.91	−19.365	< 0.001

Abbreviations: AD, Alzheimer's Disease (Aβ‐PET‐positive); HC, healthy control; MMSE, Mini‐Mental State Examination; MoCA, Montreal Cognitive Assessment.

### 3.2 Basal Forebrain Functional Connectivity Between AD and HC

Within‐group analysis revealed the specific FC pattern with Ch4 and Ch1‐3 in AD and HC groups (Figure S2). Specifically, brain regions showing positive FC with the Ch4 subregions were mainly located in the medial temporal lobe, posterior cingulate cortex/precuneus (PCC/PCUN), and some regions of the frontal and parietal lobe. Regions showing positive FC with the Ch1‐3 subregions were primarily located in the prefrontal lobe and the medial temporal lobe.

**FIGURE 1:**
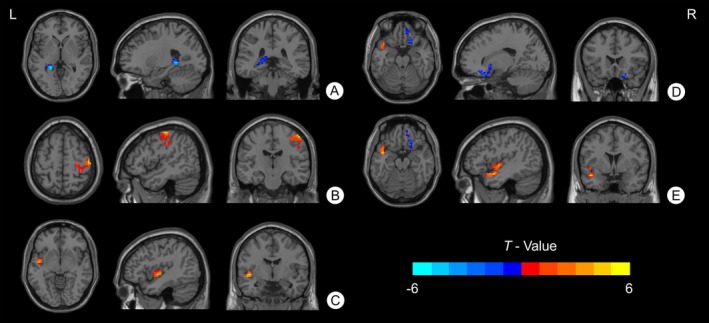
Different basal forebrain functional connectivity patterns in patients with AD compared with HC. AD patients showed (A) reduced functional connectivity between left Ch4 and the left hippocampus and the left posterior cingulate gyrus. (B) increased functional connectivity between right Ch4 and the right precentral gyrus and postcentral gyrus. (C) increased functional connectivity between left Ch1‐3 and the left superior temporal gyrus and insula. (D) reduced functional connectivity between right Ch1‐3 and the right orbital part of superior frontal gyrus and inferior frontal gyrus. (E) increased functional connectivity between right Ch1‐3 and the left superior temporal gyrus and temporal pole. *T* values are expressed in blue‐red colors from −6 to 6 (voxel‐level *p* < 0.001, cluster‐level *p* < 0.05, GRF correction).

Compared with HC, AD patients exhibited significantly decreased FC between the left Ch4 and the left hippocampus/posterior cingulate gyrus and significantly increased FC between the right Ch4 and the right precentral/postcentral gyrus. Additionally, AD patients showed increased FC between the left Ch1‐3 and the left superior temporal gyrus (STG)/insula, decreased FC between the right Ch1‐3 and the right orbital superior frontal gyrus/inferior frontal gyrus, and increased FC between the right Ch1‐3 and the left STG/temporal pole (voxel‐level *p* < 0.001, cluster‐level *p* < 0.05, GRF correction). Details are presented in Table 2 and Figure 1.

**TABLE 2:** Brain regions showing significant FC changes with Ch4 and Ch1‐3 in AD compared with HC.

Basal forebrain subregion	Cluster sizes	MNI coordinates			*t*‐value	Brain regions
		*x*	*y*	*z*		
Ch4_L	63	−21	−42	0	−5.425	Hippocampus_L/posterior cingulate gyrus_L
Ch4_R	344	51	−18	60	5.214	Precentral gyrus_R/postcentral gyrus_R
Ch1‐3_L	66	−45	−6	−3	5.327	Superior temporal gyrus_L/insula_L
Ch1‐3_R	92	18	21	−21	−4.150	Superior frontal gyrus_R/orbital part of the inferior frontal gyrus_R
	97	−45	3	−18	5.023	Superior temporal gyrus_L/superior temporal pole_L

Abbreviations: AD, Alzheimer's Disease (Aβ‐PET‐positive); HC, healthy control; L, left; MNI, Montreal Neurological Institute; *R*, right.

### 3.3 Voxel‐Wise ^18^F‐FDG PET SUVR Between AD and HC

**FIGURE 2:**
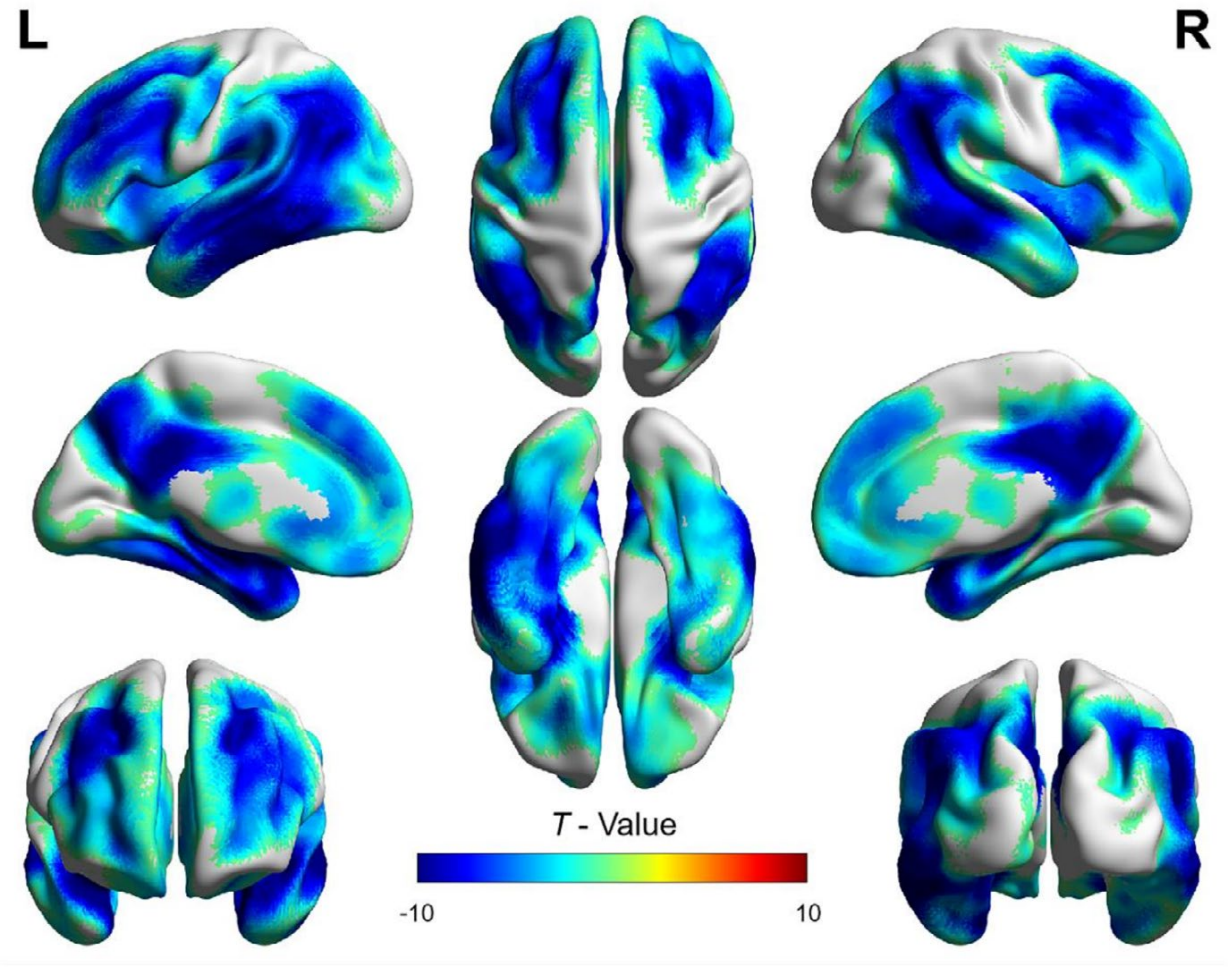
Spatial maps of the voxel‐wise analysis showing reduced ^18^F‐FDG PET SUVR with pons as the reference region in patients with AD compared with HC. *T* values are expressed in blue‐red colors from −10 to 10 (*p* < 0.05, FDR corrected).

Group differences in ^18^F‐FDG PET SUVR between AD patients and HC were assessed. The voxel‐wise analysis showed significantly reduced ^18^F‐FDG PET SUVR in the bilateral temporoparietal regions, PCC, PCUN, frontal lobes, insula, and subcortical structures including the amygdala and caudate nucleus in AD patients compared with HC (*p* < 0.05, FDR corrected) (Figure 2).

### 3.4 Spatial Correlation With Neurotransmitter Systems

#### 3.4.1 Correlation Between BF FC and Neurotransmitter Systems

**FIGURE 3:**
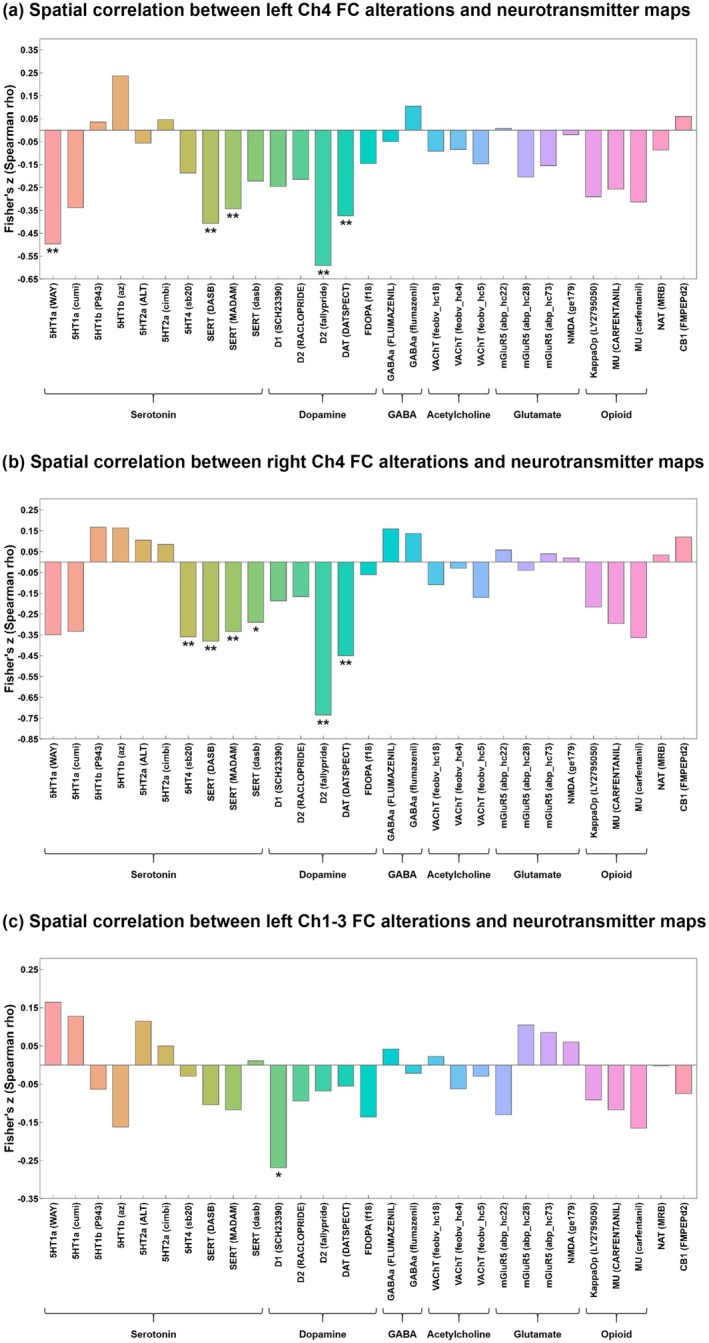
Spatial correlation between BF FC alterations (between BF subregions and the whole brain) and neurotransmitter distribution in patients with AD compared with HC (FDR corrected, *: *P*_FDR_ < 0.05, **: *p*_FDR_ < 0.01, ***: *p*_FDR_ < 0.001). AD patients showed (a) FC alterations between the left Ch4 and the whole brain were significantly negatively correlated with the spatial distribution of 5HT1a, SERT, D2, and DAT; (b) FC alterations between right Ch4 and the whole brain were significantly negatively correlated with the spatial distribution of 5HT4, SERT, D2, and DAT; (c) FC alterations between left Ch1‐3 and the whole brain were significantly negatively correlated with the spatial distribution of D1. Abbreviations: 5HT1a, 5‐hydroxytryptamine receptor subtype 1a; 5HT1b, 5‐hydroxytryptamine receptor subtype 1b; 5HT2a, 5‐hydroxytryptamine receptor subtype 2a; 5HT4, 5‐hydroxytryptamine receptor subtype 4; CB1, Cannabinoid receptor 1; D1, Dopamine D1; D2, Dopamine D2; DAT, Dopamine transporter; FDOPA, 6‐fluoro‐(^18^F)‐L‐3,4‐dihydroxyphenylalanine; GABAa, γ‐aminobutyric acid type a receptor; KappaOp, Kappa opioid receptor; mGluR5, Metabotropic glutamate receptor 5; MU, μ‐opioid receptor; NAT, Noradrenaline transporter; NMDA, N‐methyl‐D‐aspartic acid receptor; SERT, Serotonin transporter; VAChT, Vesicular acetylcholine transporter.

We employed spatial correlation analyses to examine the spatial relationships between BF subregional FC alterations and neurotransmitter maps. Compared with HC, the group‐level pattern of the left Ch4 FC alterations in AD showed a significant spatial correspondence with reference serotonergic and dopaminergic receptor and transporter maps, including 5HT1a (WAY) (*r* = −0.496, *p* = 0.003), SERT (DASB) (*r* = −0.407, *p* = 0.001), SERT (MADAM) (*r* = −0.343, *p* = 0.001), D2 (fallypride) (*r* = −0.591, *p* = 0.001), and DAT (DATSPECT) (*r* = −0.374, *p* = 0.001) (Figure 3a). Similarly, the group‐level pattern of the right Ch4 FC alterations showed a significant correspondence with reference serotonergic and dopaminergic receptor and transporter maps, including 5HT4 (sb20) (*r* = −0.359, *p* = 0.001), SERT (DASB) (*r* = −0.381, *p* = 0.001), SERT (MADAM) (*r* = −0.334, *p* = 0.005), SERT (dasb) (*r* = −0.289, *p* = 0.014), D2 (fallypride) (*r* = −0.734, *p* = 0.001), and DAT (DATSPECT) (*r* = −0.450, *p* = 0.001) (Figure 3b). In addition, the group‐level pattern of the left Ch1‐3 FC alterations showed a significant correspondence with the reference D1 (SCH23390) receptor map (*r* = −0.269, *p* = 0.035). All *p*‐values were FDR‐corrected to account for multiple comparisons (Figure 3c). No significant spatial correspondence was observed between the pattern of the right Ch1‐3 FC and any normative neurotransmitter maps after FDR correction (see uncorrected data in Table S2).

#### 3.4.2 Correlation Between ^18^F‐FDG PET SUVR and Neurotransmitter Systems

**FIGURE 4:**
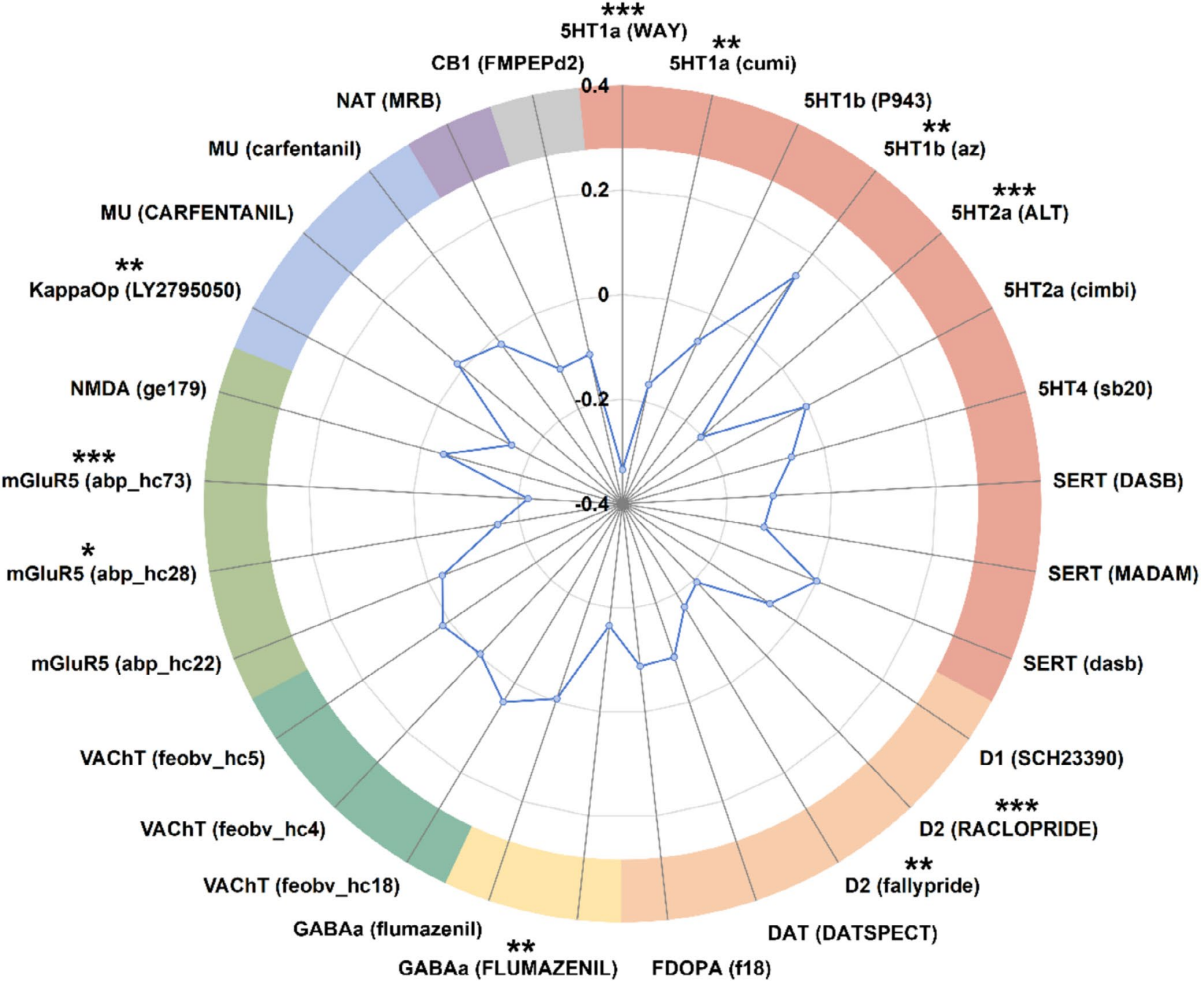
Spatial correlation between ^18^F‐FDG PET SUVR and neurotransmitter system maps in patients with AD compared with HC. The radial axis indicates correlation coefficients (FDR corrected, *: *p*_FDR_ < 0.05, **: *p*_FDR_ < 0.01, ***: *p*_FDR_ < 0.001). Abbreviations: 5HT1a, 5‐hydroxytryptamine receptor subtype 1a; 5HT1b, 5‐hydroxytryptamine receptor subtype 1b; 5HT2a, 5‐hydroxytryptamine receptor subtype 2a; 5HT4, 5‐hydroxytryptamine receptor subtype 4; CB1, Cannabinoid receptor 1; D1, Dopamine D1; D2, Dopamine D2; DAT, Dopamine transporter; FDOPA, 6‐fluoro‐(^18^F)‐L‐3,4‐dihydroxyphenylalanine; GABAa, γ‐aminobutyric acid type a receptor; KappaOp, Kappa opioid receptor; mGluR5, Metabotropic glutamate receptor 5; MU, μ‐opioid receptor; NAT, Noradrenaline transporter; NMDA, N‐methyl‐D‐aspartic acid receptor; SERT, Serotonin transporter; VAChT, Vesicular acetylcholine transporter.

We performed spatial correlation analyses to examine whether ^18^F‐FDG PET SUVR alterations in AD patients showed spatial overlap with the reference distribution patterns of specific neurotransmitter systems (*p*_FDR_ < 0.05, Figure 4). Compared with HC, ^18^F‐FDG PET SUVR alterations in AD patients showed significant negative correspondence with several normative neurotransmitter maps, including 5HT1a (WAY) (*r* = −0.335, *p* < 0.001), 5HT1a (cumi) (*r* = −0.166, *p* = 0.007), 5HT2a (ALT) (*r* = −0.204, *p* < 0.001), D2 (RACLOPRIDE) (*r* = −0.194, *p* < 0.001), D2 (fallypride) (*r* = −0.170, *p* = 0.001), GABAa (FLUMAZENIL) (*r* = −0.166, *p* = 0.002), mGluR5 (abp_hc28) (*r* = −0.158, *p* = 0.017), mGluR5 (abp_hc73) (*r* = −0.219, *p* < 0.001), and KappaOp (LY2795050) (*r* = −0.160, *p* = 0.002). A significant positive correspondence was observed between glucose hypometabolism and the reference 5HT1b (az) map (*r* = 0.147, *p* = 0.003).

## 4 Discussion

This study demonstrated that alterations in resting‐state FC of BF subregions and cerebral glucose metabolism in AD exhibited distinct spatial correspondences with reference neurotransmitter receptor and transporter maps. Specifically, the spatial patterns of BF FC alterations overlapped with cortical regions characterized by high densities of serotonergic and dopaminergic receptors in normative maps, whereas the topography of cerebral hypometabolism showed a broader pattern of spatial correspondence including serotonergic, dopaminergic, GABAergic, glutamatergic, and kappa‐opioid systems. These findings support our hypothesis that BF‐related network and cerebral metabolic changes in AD co‐localize with specific neurotransmitter maps, providing new insights into the potential neurochemical underpinnings of AD pathology and informing potential therapeutic strategies in AD.

Previous resting‐state fMRI study in healthy adult individuals has demonstrated a functional segregation within BF subregions, with Ch1‐3 (medial septum and vertical limb of the diagonal band) coupled to the hippocampus and medial cortical memory‐related regions, whereas Ch4 (nucleus basalis of Meynert) shows stronger connectivity with salience and attention‐related cortical areas. Although hippocampal connectivity is more prominently expressed in Ch1‐3, Ch4‐related regions participate in cortico‐hippocampal and default mode network interactions at the network level [41]. In this context, we observed decreased FC between the left Ch4 and the left hippocampal/posterior cingulate gyrus in patients with AD, indicating the crucial role of NbM in modulating limbic and default mode networks in AD. Core regions of the default mode network exhibit the most pronounced dysfunction across the AD continuum, particularly in the PCC, PCUN, inferior parietal cortex, and hippocampus. Consistent with our findings, a previous fMRI study has similarly reported reduced NbM‐FC in the hippocampus from early MCI to late MCI [16]. A selective immunotoxin lesion study in APP/PS1 mice demonstrated that targeted loss of basal forebrain cholinergic neurons (including NbM) removes approximately 50% of cortical and hippocampal cholinergic innervation, contributing to impairments in learning and memory [43]. Longitudinal structural MRI studies have shown that atrophy of the posterior Ch4 and hippocampus, but not Ch1/Ch2, is tightly associated with early memory and attention decline in preclinical and prodromal AD [44], and NbM serves as an important predictor of hippocampal and cortical degeneration [7, 45]. These findings suggest that AD pathology preferentially compromises both structural and functional integrity of memory‐related circuits. This selective vulnerability underscores the pivotal role of Ch4 projections in sustaining large‐scale cognitive networks.

Interestingly, our results also showed increased FC between the right Ch4 and the precentral and postcentral gyri of the somatosensory network in patients with AD. Such hyperconnectivity may reflect a stage‐dependent reorganization of cortical networks in AD. Sensorimotor cortex is located at the high structural‐functional “tethering” end of the BF connectivity gradient, indicating that its coupling with BF is more strongly constrained by direct structural pathways [46]. This anatomical property suggests that sensorimotor systems may remain relatively preserved and participate in large‐scale functional reconfiguration. Task‐based fMRI evidence reported increased activation of the postcentral gyrus and head of the caudate in amnestic MCI, which was associated with hippocampal atrophy and disease duration, whereas this over‐engagement was not preserved in AD, indicating that early increases may diminish with disease progression [47]. A resting‐state fMRI study demonstrated widespread FC increases in patients with mild AD compared with HC, involving motor‐ and sensorimotor‐related regions, and interpreted these effects as reflecting dysregulated and non‐linear connectivity emerging in the context of neurodegeneration [48]. Our findings suggest that as memory‐related default mode circuits undergo progressive disconnection, intrinsic connectivity patterns may shift toward sensorimotor systems that are relatively preserved structurally, reflecting a redistribution of large‐scale network interactions.

We further observed that AD patients showed heterogeneous FC alterations related to the Ch1‐3, including increased connectivity between the left Ch1‐3 and STG/insula, reduced connectivity between the right Ch1‐3 and orbitofrontal cortices, and enhanced cross‐hemispheric connectivity with the left STG/temporal pole. The orbitofrontal cortex is essential for reward evaluation and goal‐directed behavior. The STG is involved in passive storage and active retrieval of semantic information [49], is vulnerable to tau deposition, and receives dense cholinergic projections from the BF, which provide an anatomical substrate for neuromodulatory control of temporal cortical processing [10]. The insula serves as a central hub for emotional and cognitive integration. The mid‐cingulate‐insula network exhibits the lowest structural–functional “tethering” and the highest density of cholinergic terminals, making it particularly vulnerable to excitation/inhibition imbalance and disinhibition in AD. Previous studies have shown that degeneration of the BF can disrupt cortical excitation–inhibition balance, which may facilitate disinhibition‐driven synchronization and aberrant coupling between large‐scale networks at rest [50]. A plausible explanation for this finding is that reduced Ch1‐3–orbitofrontal connectivity in AD may reflect impaired cholinergic modulation of the motivation–executive control system, with concomitant shifts in intrinsic connectivity toward temporal–insular regions.

The spatial correspondence between group‐level patterns of BF subregional FC alterations and monoaminergic reference maps suggests that AD‐related BF network changes tend to spatially overlap with cortical regions characterized by higher serotonergic and dopaminergic receptor or transporter density. The serotonergic and dopaminergic systems are involved in memory and cognitive functions, and their dysfunction has been increasingly explored in AD [21, 26]. Lower serotonin transporter levels have been observed in individuals with MCI, which correlate with memory deficits [21]. Similarly, dopaminergic dysfunction, including reduced D2 receptor availability and dopamine transporter loss, has been linked to apathy, executive deficits, and impaired reward processing [26]. Multimodal meta‐analytic evidence has reported that AD patients exhibit structural and functional alterations in the PCC/PCUN and parahippocampal gyrus, which are associated with serotonergic and dopaminergic system degeneration [51]. Within this framework, the observed spatial overlap may reflect a shared regional vulnerability of highly integrated, monoaminergically modulated networks to AD pathology.

Glucose metabolism serves as the indispensable fuel of the brain and engages in reciprocal regulation with neurotransmitter systems, jointly sustaining cognitive functions and neurobiological homeostasis. In our study, we observed that the topography of hypometabolism, which included temporoparietal cortices, PCC, PCUN, frontal regions, insula, and subcortical structures, and was consistent with the canonical metabolic pattern of AD [52], showed significant negative spatial correlations with reference densities of 5HT1a, 5HT2a, D2, GABAa, mGluR5, and kappa‐opioid receptors. This finding indicates that brain regions with higher densities of serotonergic, dopaminergic, GABAergic, glutamatergic, and kappa‐opioid systems in healthy brains tend to exhibit more pronounced hypometabolism in AD. This pattern may reflect a selective vulnerability of highly receptor‐dense cortical territories to AD‐related neurodegenerative processes, a hypothesis that aligns with the recent study linking neurotransmitter system distributions to patterns of neurodegeneration [36, 53].

This is the first study to investigate the spatial correlations between BF subregional FC, cerebral glucose metabolism and normative neurotransmitter system maps in AD. From a clinical perspective, our findings have several implications. Although the cholinergic system plays a key role in AD, our results indicate that the spatial patterns of serotonergic, dopaminergic, GABAergic, and glutamatergic system distributions in the healthy brain correspond to regions showing functional and metabolic alterations in AD, highlighting the potential involvement of multiple neurotransmitter systems in the pathophysiology of the disease. Moreover, our observations support the rationale for multi‐target therapeutic strategies. While current cholinesterase inhibitors provide modest benefits but fail to halt disease progression, interventions targeting serotonergic or dopaminergic modulation may provide complementary or synergistic effects.

Our study has several limitations. First, the restricted sample size and retrospective nature necessitate larger cohorts with prospective follow‐up to enhance reliability. Second, the lack of participants with MCI and subjective cognitive decline constrained validation of our results across the entire AD spectrum. Third, amyloid PET was not performed in healthy control participants, and future studies should include biomarker‐confirmed Aβ‐negative controls to validate our findings. Fourth, the neurotransmitter maps were derived from healthy cohorts and represent normative receptor/transporter distributions; therefore, our findings reflect spatial correspondences with these reference maps and do not provide direct information about receptor status or synaptic integrity in our patient sample. Fifth, since tau pathology is more closely linked to neurodegeneration and cognitive decline, future multimodal studies incorporating tau‐PET will be crucial to disentangle the relationships between AD pathology, functional/metabolic changes, and neurotransmitter system distributions.

## 5 Conclusions

This study demonstrated that BF subregional FC alterations and widespread cortical glucose hypometabolism in AD show distinct spatial correspondences with normative maps of specific neurotransmitter systems, particularly serotonergic and dopaminergic pathways. These findings highlight potential neuroanatomical links between BF‐related network dysfunction and brain regions rich in these neuromodulatory systems. Future longitudinal and multimodal studies are warranted to elucidate the causal relationships underlying these spatial correspondences to uncover their role in disease progression, and to explore their translational potential for early diagnosis and therapeutic intervention.

## Author Contributions

Conceptualization: Yujie He and Shaozhen Yan. Methodology: Yujie He, Hanxiao Xue, and Sheng Bi. Formal analysis: Yujie He and Hanxiao Xue. Investigation: Yujie He, Hanxiao Xue, Sheng Bi. Resources: Shaozhen Yan and Jie Lu. Data curation: Yujie He, Hanxiao Xue, Sheng Bi, Yan Wang, Xiaocao Liu, Yixia Li, Zhigeng Chen, Dongdong Rong, Bixiao Cui, and Jie Ma. Writing – original draft preparation: Yujie He. Writing – review and editing: Shaozhen Yan and Jie Lu. Supervision: Shaozhen Yan and Jie Lu. Project administration: Shaozhen Yan and Jie Lu. Final approval of manuscript: All authors.

## Funding

This work was supported by National Natural Science Foundation of China (82394434, 62333002), Xuanwu Hospital Talent Convergence Program (HZ2025PYDTR006).

## Disclosure

The authors have nothing to report.

## Ethics Statement

The study was approved by the Research Ethics Review Board of Xuanwu Hospital (2022[078]) and conducted in accordance with the Declaration of Helsinki. All participants provided written informed consent.

## Conflicts of Interest

The authors declare no conflicts of interest.

## Supporting information


**Figure S1:** Location of the basal forebrain (BF) seed regions used for functional connectivity analysis. Axial and coronal sections illustrate the BF subregions defined as Ch1‐3 and Ch4 in standard MNI space. The Ch1‐3 region (shown in green) comprises the medial septal nucleus and the vertical and horizontal limbs of the diagonal band. The Ch4 region (shown in red) corresponds to the nucleus basalis of Meynert.
**Figure S2:** Basal forebrain subregional functional connectivity network maps in Alzheimer's disease (AD) and healthy control (HC) groups. Spatial patterns of positive seed‐to‐voxel functional connectivity for the left and right Ch4 and Ch1‐3 subregions of the basal forebrain in the two groups (*p* < 0.001, FDR corrected). The color bar indicates *t* values.
**Table S1:** Functions of neurotransmitter receptors or transporters included in JuSpace toolbox.
**Table S2:** Spatial correlations between basal forebrain subregional functional connectivity alterations (the right Ch1‐3) and neurotransmitter receptor/transporter maps (uncorrected *p*‐values).

## Data Availability

The data that support the findings of this study are available from the corresponding author upon reasonable request.
