# Supplementary material for: Correspondence of Basal Forebrain Resting‐State Functional Connectivity and Cerebral Glucose Metabolism Alterations With Neurotransmitter Maps in Alzheimer's Disease
Source: CNS Neurosci Ther. 2026 May 9;32(5):e70901. doi: 10.1002/cns.70901 (PMC13156800; doi:10.1002/cns.70901)
Supplement: Supplementary file 1 — Figure S1: Location of the basal forebrain (BF) seed regions used for functional connectivity analysis. Axial and coronal sections illustrate the BF subregions defined as Ch1‐3 and Ch4 in standard MNI space. The Ch1‐3 region (shown in green) comprises the medial septal nucleus and the vertical and horizontal limbs of the diagonal band. The Ch4 region (shown in red) corresponds to the nucleus basalis of Meynert. Figure S2: Basal forebrain subregional functional connectivity network maps in Alzheimer's disease (AD) and healthy control (HC) groups. Spatial patterns of positive seed‐to‐voxel functional connectivity for the left and right Ch4 and Ch1‐3 subregions of the basal forebrain in the two groups (p < 0.001, FDR corrected). The color bar indicates t values. Table S1: Functions of neurotransmitter receptors or transporters included in JuSpace toolbox. Table S2: Spatial correlations between basal forebrain subregional functional connectivity alterations (the right Ch1‐3) and neurotransmitter receptor/transporter maps (uncorrected p‐values). [file CNS-32-e70901-s001.docx]

Supplementary materials for the manuscript titled “Correspondence of Basal Forebrain Resting-State Functional Connectivity and Brain Glucose Metabolism Alterations with Neurotransmitter Maps in Alzheimer’s Disease”.

This file (*Supplementary Material.docx*) contains two supplementary figures and two supplementary tables.

**
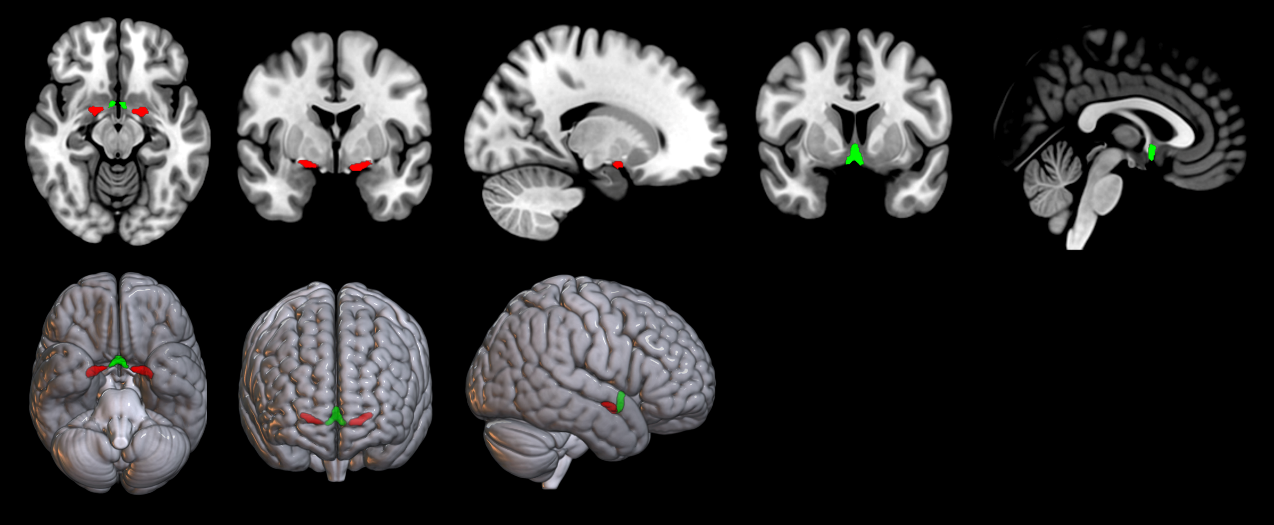
**

Figure S1. Location of the basal forebrain (BF) seed regions used for functional connectivity analysis. Axial and coronal sections illustrate the BF subregions defined as Ch1-3 and Ch4 in standard MNI space. The Ch1-3 region (shown in green) comprises the medial septal nucleus and the vertical and horizontal limbs of the diagonal band. The Ch4 region (shown in red) corresponds to the nucleus basalis of Meynert.


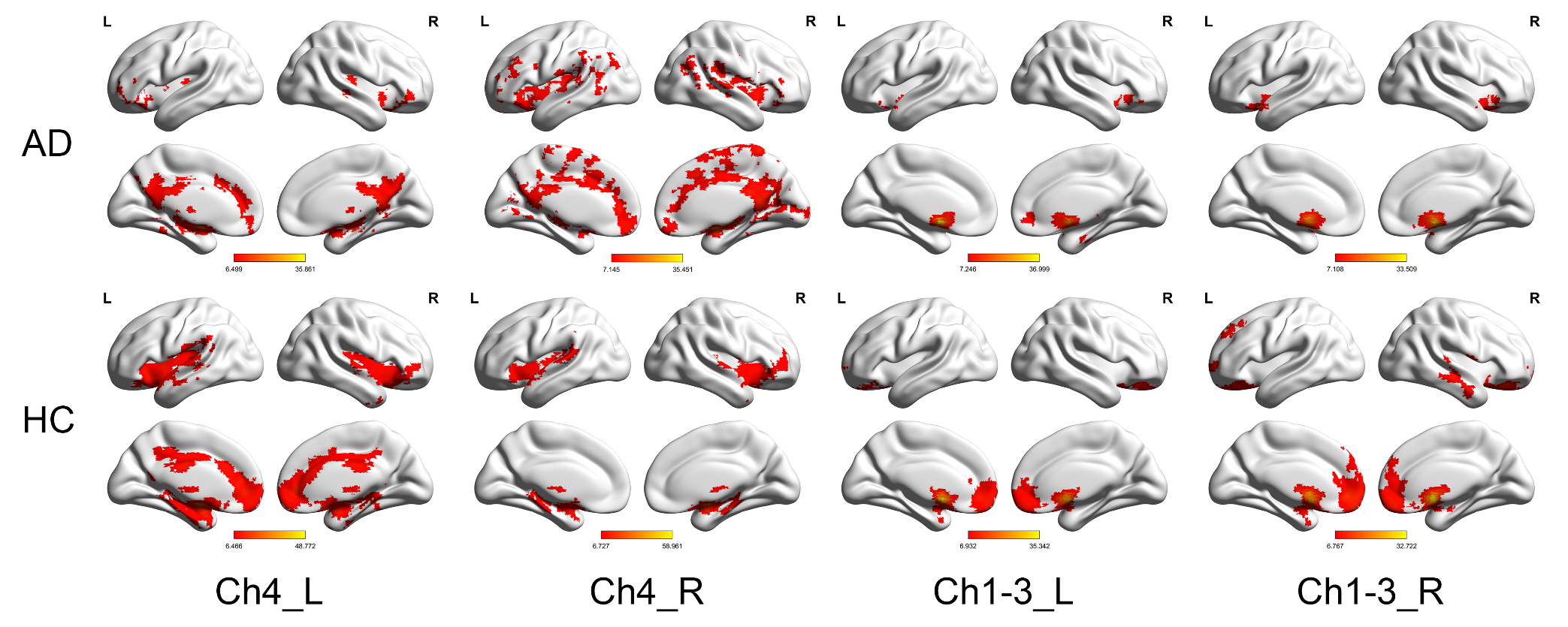


Figure S2. Basal forebrain subregional functional connectivity network maps in Alzheimer’s disease (AD) and healthy control (HC) groups. Spatial patterns of positive seed-to-voxel functional connectivity for the left and right Ch4 and Ch1-3 subregions of the basal forebrain in the two groups (*P*<0.001, FDR corrected). The color bar indicates *t* values.

**Table S1. Functions of neurotransmitter receptors or transporters included in JuSpace toolbox.**

| **Neurotransmitter receptor/transporter** | | **Maps** | **Radioligands** | **Functions** |
| --- | --- | --- | --- | --- |
| **Serotonin** | **5HT1a** | 5HT1a_WAY_HC36  5HT1a_cumi | [^11^C]WAY-100635  [^11^C]CUMI-101 | Emotion regulation, hippocampal plasticity, memory |
|  | **5HT1b** | 5HT1b_P943_HC22  5HT1b_az_hc36_beliveau | [^11^C]P943  [^11^C]AZ10419369 | motor control, reward, anxiety modulation |
|  | **5HT2a** | 5HT2a_ALT_HC19  5HT2a_cimbi_hc29 | [^18^F]ALT  [^11^C]Cimbi-36 | Cortical excitation, cognitive control, perception, stress detection |
|  | **5HT4** | 5HT4_sb20_hc59 | [^11^C]SB207145 | Memory enhancement, cholinergic facilitation |
|  | **SERT** | SERT_DASB_HC30  SERT_MADAM_c11  SERT_dasb_hc100 | [^11^C]DASB  [^11^C]MADAM  [^11^C]DASB | Serotonin reuptake, mood regulation, control in motor impulsivity |
| **Dopamine** | **D1** | D1_SCH23390_c11 | [^11^C]SCH23390 | cognition, motivation and addiction |
|  | **D2** | D2_RACLOPRIDE_c11  D2_fallypride_hc49 | [^11^C]Raclopride  [^18^F]Fallypride | Striatal plasticity, motor control, reward |
|  | **DAT** | DAT_DATSPECT | [^123^I]FP-CIT | Dopamine clearance, motor behavior |
|  | **FDOPA** | FDOPA_f18 | [^18^F]FDOPA | Dopamine synthesis capacity, motor behavior |
| **Glutamate** | **mGlu5** | mGluR5_abp_hc22  mGluR5_abp_hc28  mGluR5_abp_hc73 | [^11^C]ABP688  [^11^C]ABP688  [^11^C]ABP688 | Excitatory signaling, synaptic plasticity, learning and memory |
|  | **NMDA** | NMDA_ge179_29hc | [^18^F]GE179 | Learning, long-term potentiation, excitatory drive |
| **GABA** | **GABAa** | GABAa_FLUMAZENIL_c11  GABAa_flumazenil_hc16 | [^11^C]Flumazenil  [^11^C]Flumazenil | Cortical inhibition, network stability, learning and memory formation, sensory-motor control |
| **Acetylcholine** | **VAChT** | VAChT_feobv_hc18  VAChT_feobv_hc4  VAChT_feobv_hc5 | [^18^F]FEOBV  [^18^F]FEOBV  [^18^F]FEOBV | Cholinergic transmission, attention, memory, regulation of cortical activity and cerebral blood flow |
| **Opioids** | **Kappa** | KappaOp_LY2795050_hc10 | [^11^C]LY2795050 | Stress regulation, dysphoria, pain modulation |
|  | **MU** | MU_CARFENTANIL_c11  MU_carfentanil_hc39 | [^11^C]Carfentanil  [^11^C]Carfentanil | Reward, affective tone, analgesia |
| **Noradrenaline** | **NAT** | NAT_MRB_c11 | [^11^C]MRB | Arousal, vigilance, attention control |
| **Cannabinoid** | **CB1** | CB1_FMPEPd2_hc22 | [^18^F]FMPEP-d2 | Synaptic modulation, emotional regulation |

Abbreviations: 5HT1a: 5-hydroxytryptamine receptor subtype 1a, 5HT1b: 5-hydroxytryptamine receptor subtype 1b, 5HT2a: 5-hydroxytryptamine receptor subtype 2a, 5HT4: 5-hydroxytryptamine receptor subtype 4, SERT: serotonin transporter, D1: dopamine D1, D2: dopamine D2, DAT: dopamine transporter, FDOPA: 6-fluoro-(^18^F)-L-3,4-dihydroxyphenylalanine, GABAa: γ-aminobutyric acid type a receptor, VAChT: vesicular acetylcholine transporter, mGluR5: metabotropic glutamate receptor 5, NMDA: N-methyl-D-aspartic acid receptor, KappaOp: kappa opioid receptor, MU: μ-opioid receptor, NAT: Noradrenaline transporter, CB1: Cannabinoid receptor 1.

**Table S2. Spatial correlations between basal forebrain subregional functional connectivity alterations (the right Ch1-3) and neurotransmitter receptor/transporter maps (uncorrected *P*-values).**

| Neurotransmitter System | Radioligand (Atlas Name) | Correlation Coefficient (*r*) | Uncorrected *P*-value | Corrected *P*-value |
| --- | --- | --- | --- | --- |
| **Serotonergic** | 5HT1a (WAY_HC36) | 0.256 | 0.048* | 0.220 |
|  | 5HT1a (cumi_hc8_beliveau) | 0.151 | 0.276 | 0.661 |
|  | 5HT1b (P943_HC22) | -0.094 | 0.537 | 0.816 |
|  | 5HT1b (az_hc36_beliveau) | -0.231 | 0.012* | 0.080 |
|  | 5HT2a (ALT_HC19) | 0.152 | 0.097 | 0.366 |
|  | 5HT2a (cimbi_hc29_beliveau) | 0.053 | 0.711 | 0.880 |
|  | 5HT4 (sb20_hc59_beliveau) | -0.034 | 0.720 | 0.880 |
|  | SERT (DASBHC30) | -0.048 | 0.772 | 0.882 |
|  | SERT (MADAM_c11) | 0.135 | 0.334 | 0.706 |
|  | SERT (dasb_hc100_beliveau) | -0.238 | 0.010* | 0.068 |
| **Dopaminergic** | D1 (SCH23390_c11) | -0.084 | 0.370 | 0.706 |
|  | D2 (RACLOPRIDE_c11) | 0.014 | 0.870 | 0.946 |
|  | D2 (fallypride_hc49_jaworska) | -0.040 | 0.660 | 0.876 |
|  | DAT (DATSPECT) | -0.200 | 0.027* | 0.150 |
|  | FDOPA (f18) | 0.075 | 0.420 | 0.776 |
| **GABAergic** | GABAa (FLUMAZENIL_c11) | -0.075 | 0.634 | 0.876 |
|  | GABAa (flumazenil_hc16) | -0.055 | 0.735 | 0.881 |
| **Glutamatergic** | mGluR5 (abp_hc22_rosanetom) | -0.089 | 0.664 | 0.876 |
|  | mGluR5 (abp_hc28_duboism) | -0.138 | 0.537 | 0.816 |
|  | mGluR5 (abp_hc73_smart) | -0.007 | 0.941 | 0.966 |
|  | NMDA (ge179_29hc) | -0.008 | 0.949 | 0.966 |
| **Cholinergic** | VAChT (feobv_hc18_aghourian) | -0.094 | 0.311 | 0.691 |
|  | VAChT (feobv_hc4_tuominen) | -0.100 | 0.283 | 0.666 |
|  | VAChT (feobv_hc5_bedardm) | -0.020 | 0.829 | 0.912 |
| **Others** | CB1 (FMPEPd2_hc22) | -0.003 | 0.977 | 0.985 |
|  | KappaOp (LY2795050_hc10) | -0.096 | 0.296 | 0.683 |
|  | MU (CARFENTANIL_C11) | -0.050 | 0.588 | 0.838 |
|  | MU (carfentanil_hc39_turtonen) | -0.092 | 0.533 | 0.816 |
|  | NAT (MRB_c11) | 0.142 | 0.347 | 0.706 |
